# Survey design for broad-scale, territory-based occupancy monitoring of a raptor: Ferruginous hawk (*Buteo regalis*) as a case study

**DOI:** 10.1371/journal.pone.0213654

**Published:** 2019-03-22

**Authors:** Tracey N. Johnson, Kristen Nasman, Zachary P. Wallace, Lucretia E. Olson, John R. Squires, Ryan M. Nielson, Patricia L. Kennedy

**Affiliations:** 1 Department of Fish and Wildlife Sciences, University of Idaho, Moscow, Idaho, United States of America; 2 Western Ecosystems Technology, Inc., Cheyenne, Wyoming, United States of America; 3 Wyoming Natural Diversity Database, University of Wyoming, Laramie, Wyoming, United States of America; 4 Rocky Mountain Research Station, U.S. Forest Service, Missoula, Montana, United States of America; 5 Department of Fisheries and Wildlife and Eastern Oregon Agriculture Research Center, Oregon State University, Union, Oregon, United States of America; University of Alberta, CANADA

## Abstract

Given the uncertain population status of low-density, widely-occurring raptors, monitoring changes in abundance and distribution is critical to conserving populations. Nest-based monitoring is a common, useful approach, but the difficulty and expense of monitoring raptor nests and importance of reliable trend data to conservation requires that limited resources are allocated efficiently. Power analyses offer a helpful tool to ensure that monitoring programs have the ability to detect trends and to optimize financial resources devoted to monitoring. We evaluated alternative monitoring designs for raptors to identify appropriate survey effort to detect population trends. We used data collected from a territory-occupancy study of ferruginous hawks throughout Wyoming to guide simulations and evaluate the ability to detect trends in occupancy rates. Results suggest that greater gains in precision of trend estimation may be achieved through the addition of more sites and not more visits; statistical power was ≥80% when monitoring lasted 20 years and population declines were 20%; and probability of detection affected statistical power less than rates of population decline. Monitoring at least 150 sites for 20 years would provide reasonable estimates of trend in occupancy given certain rates of detection and occupancy, but only for population declines of 20%. Removal sampling did not result in substantial changes of any metrics used to evaluate simulations, providing little justification for employing the standard design if territory occupancy is the variable of interest. Initial rates of territory occupancy may be biased high, a problem inherent to many studies that monitor territory occupancy. We explored the effects of lower rates of initial occupancy on the ability to detect trends. Although we present data from a study of ferruginous hawks, our simulations can be applied to other raptor species with similar life history and population dynamics to provide guidance for future trend estimation of territory occupancy.

## Introduction

Anthropogenic transformation of natural systems is currently a primary driver of species abundance and distribution patterns, leading to population declines for many species [1, 2]. Consequences are of particular concern for species that are already rare, especially those that occupy a high trophic position, restricted geographic ranges, and narrow environmental gradients [3]. Apex predators, such as raptors, often meet criteria for rarity and consequently, many species are of conservation concern. Presently, the International Union for Conservation of Nature (IUCN) reports 86 species within Order: Accipitriformes (34%) as either Critically Endangered, Endangered, Vulnerable, or Near Threatened [4]. In the United States, ten raptor species (45%) within the same order have been designated as species of conservation concern in the last decade [5]. Raptors fill unique ecosystem roles as indicators of environmental pollutants [6, 7], flagship species [8], and apex predators responsible for trophic cascades [9, 10]. Given the implications of their loss from ecological communities to ecosystem functioning, it follows that there is substantial interest in their conservation and management from regional to global scales (e.g., Hawks Aloft, Inc., Hawk Mountain Sanctuary, Hawkwatch International, The Peregrine Fund, and many others).

Human alteration of landscapes is a primary threat to many raptor species. Direct mortality results from collisions with human infrastructure and electrocution [11–14], and reproductive success can be reduced by changes in land-use or climate that alter prey availability or predation risk of young [15]. In the western U.S., some populations of raptors have experienced declines since the 1990s [15]. In particular, raptors occupying sagebrush steppe and grasslands have experienced extensive landscape changes from accelerated habitat conversion driven by agriculture, urban, rural, and energy sprawl, and altered fire regimes [16–18]. For species like ferruginous hawks (*Buteo regalis*) whose geographic distribution occurs entirely within western North America, these landscape alterations affect the majority of the population [19, 15].

Given the uncertain population status of low-density, widely occurring species, monitoring changes in both the abundance and distribution of raptors will be critical to conserving their populations. Bald eagles (*Haliaeetus leucocephalus*) and golden eagles (*Aquila chrysaetos*), along with northern goshawks (*Accipiter gentilis*), each have rigorous monitoring programs in the U.S. [20–22]. However, other special-status species such as ferruginous hawks lack standardized monitoring programs; this is likely affected by their broad range and varying conservation designations across multiple management entities. Further, many raptor species are not well-captured by multi-species monitoring programs such as the North American Breeding Bird Survey or Integrated Monitoring in Bird Conservation Regions because surveys used by such programs typically yield relatively few observations of raptors, making precise or accurate abundance or trend estimation difficult [23, 24]. Thus, there is a need for the development of standardized monitoring for ferruginous hawks and similar species.

The low density of many raptor populations, combined with their high fidelity to territories and nest sites, and the conspicuousness of their large stick nests, has resulted in monitoring focused on known (historic) nests or territories e.g., [25–28]. Nest-based monitoring may also be preferred over alternative approaches, like counting individuals, because of the additional benefit of acquiring information on reproductive success [29]. However, nest monitoring for rare species across broad scales poses unique challenges. For extensive and remote survey areas, nest monitoring is commonly conducted using aircraft [24, 30], but permitting (for eagles), airspace regulations, seasonal weather patterns, safety, and cost must be accounted for. Further, apparent occupancy of nests or territories can vary dramatically among years, either caused by variable detection rates [27, 31], or variability in true nest site occupancy and reproductive output, often in response to climatic conditions or fluctuating prey populations [32]. Thus, sample sizes must be large enough to detect trends despite potentially high interannual variability, and appropriate methods must be used to account for imperfect detection.

Methods for estimating site occupancy rates, for example proportion of nests or territories occupied for breeding, are employed when there is interest in occurrence-based indices of species but imperfect detection of individuals [33]. MacKenzie et al. [34, 33] described a method of modeling site occupancy that allows estimation of occupancy rates when occupancy status of a nest changes among seasons during a study period (i.e., colonization or local extinction). Given the difficulty and expense of monitoring raptor nests and the importance of representative trend data for management and conservation of raptor populations, power analyses offer a valuable tool to ensure that monitoring programs have the ability to detect trends and to optimize financial resources devoted to monitoring. Power analyses are especially useful in guiding the development of monitoring programs for rare animals [35–37], but only recently have they been extended to include an occupancy framework [38–43]. Although occupancy estimation has been used for raptor monitoring programs (e.g., northern goshawk [20]; Mexican spotted owl (*Strix occidentalis lucida*; [44]), use of power analysis to guide the development of such programs is lacking for most raptor species (but see [36]), even though the financial and biological cost of conducting a program with inadequate power could be high.

Here, our objective is to provide practical guidance on effort and sample designs based on simulations of population parameters and multiple aspects of a territory-occupancy based monitoring program. We use ferruginous hawks as a case study because this species is dependent on sagebrush-dominated ecosystems that are increasingly impacted by anthropogenic change. Furthermore, we lack standardized monitoring protocols for this species given the difficulty in assessing its population status, and we have available a dataset of territory occupancy for ferruginous hawks across Wyoming, U.S.A. that provided an ideal framework to evaluate power analyses as they relate to guiding the development of raptor monitoring programs. We use simulations to evaluate alternative designs for monitoring given a range of values for survey effort and population-level parameters that may influence the power to detect population trends. We consider multiple aspects of survey effort in order to achieve specific monitoring objectives, including: the number of sites to be monitored, how many surveys per site to conduct within a season, and total duration of the monitoring program. We examine two approaches to multi-season occupancy estimation: a standard site-occupancy design which includes an equal number of visits among all sampling units (e.g., nesting territories) throughout the breeding season, and a removal design in which surveys are discontinued (within the respective breeding season only) after detection of the target species. A removal design may improve efficiency and reduce cost through fewer visits to some sites within a season. The analysis presented here was facilitated by a need for guidance on the development of a territory-occupancy monitoring program for ferruginous hawks throughout the state of Wyoming, but the simulations and other considerations we present can provide guidance for monitoring other raptors for which estimation of territory-occupancy is of interest at broad scales. Based on these simulations, we offer recommendations on sample design and effort while considering logistical constraints.

## Materials and methods

Briefly, we used data presented in [27] to guide simulations allowing us to evaluate the ability to detect trends in occupancy rates for ferruginous hawks. We evaluated trends in occupancy over a time period of 10 or 20 years given various characteristics of the ferruginous hawk population and survey effort. Wallace et al. [27] reported data collected from territory-based detection-non detection surveys in Wyoming during the ferruginous hawk breeding season from 2011–2013. The authors present rates of re-occupancy of known territories estimated from single-season models and related to environmental covariates including oil and gas infrastructure, sagebrush cover, nest substrate, and prey populations. Using the same data presented in Wallace et al. [27], we simulated trends in territory occupancy for ferruginous hawks using a multi-season site-occupancy approach [33]. We identified distributions for initial probability of occupancy, probability of detection, and probability of colonization and extinction, and evaluated the ability to detect trends using multiple statistical metrics described below.

### Study area

The study area at which data were collected that formed the basis for simulations included 1,230 townships comprising the range of ferruginous hawks in Wyoming [45, 27]. Townships were delineated by the U.S. Public Land Survey System and are approximately square with sides 9.66 km in length [46]. The sample units were putative breeding territories within the townships, defined as circular buffers with a 1.5-km radius around nest sites historically occupied by ferruginous hawk that were located during systematic aerial surveys of townships [45, 27].

### Simulation design

To evaluate the ability to detect trends in occupancy of ferruginous hawks under different scenarios we developed simulations that varied: 1) survey effort, including number of monitored territories, number of visits to a territory within a breeding season, and total duration of monitoring (i.e., number of years), 2) the observation process (probability of detection; *p*), and 3) parameters associated with territory occupancy, including initial proportion of sites occupied [*ψ*_*1*_], and annual rates of decline in regional ferruginous hawk occupancy. We conducted simulations using a standard sampling design in which all sampling units are visited an equal number of times, and a removal sampling design in which visits to sampling units cease (within the current breeding season) once presence is confirmed. We arrived at the values used for simulations (Table 1) with the approach described below.

**Table 1:** Parameters and simulated values used in simulations of territory-occupancy monitoring of ferruginous hawks.

Parameter	Simulated values
*Survey effort*	
Number of sites	100–200
Number of visits	2–6 per season^a^
Total duration	10 yrs; 20 yrs
*Observation process*	
Probability of detection	0.44; 0.67; 0.79
*Occupancy*	
Probability of extinction	0.33 (fixed)
Initial probability of occupancy	0.42; 0.57; 0.72
Annual population decline	1.12%; 0.55%^b^

^a^ For the standard sampling design, this value represents the total number of visits to a given site per season. For the removal sampling design, this value represents the maximum number of visits to a given site per season.

^b^ Annual decreases represent a 20% and 10% decline over 20 years, respectively.

The range of values for survey effort was based on exploratory simulations and logistical and financial constraints identified during previous survey efforts [27]. Initial simulations suggested that dropping the minimum number of sites to 75 (below that used in Wallace et al. [27]) would not provide adequate statistical power. Thus, the number of sampled sites we chose was simulated to range from 100–200, 100 representing the approximate sample size used in [27] and 200 representing the maximum number of sites that can be sampled within one breeding season using aerial survey methods given similar budget constraints. The number of visits we simulated ranged from two to six, two representing the minimum number of visits required to estimate detection probability when detection is imperfect [33] and six representing the maximum number of visits that could reasonably be conducted within one season using the removal design, assuming detection rates within the range of those reported in [27]; see below; [33] and a trade-off with number of sampled sites.

The range of values we used for probability of detection (*p*) was reported in [27]. In 2011, 2012, and 2013, $\hat{p}$ was 0.79, 0.78, and 0.44, respectively [27]. We assumed the highest and lowest values of $\hat{p}$ (i.e., 0.79 and 0.44) within the simulation to evaluate the influence of high and low detection rates on the power to detect trends in occupancy. Additionally, we used the medium value for $\hat{p}$ of 0.67 (average of 2011, 2012, and 2013). We simulated probability of extinction (*ε*) at a fixed value of 0.33, which we estimated from a multi-season occupancy model fit to the data presented in [27].

Managers tasked with monitoring ferruginous hawks may want the ability to detect relatively small declines in occupancy to prepare management or mitigation responses should the trend continue. Thus, we explored the ability to detect relatively small annual decreases in the ferruginous hawk population (π) of 0.55% and 1.12% (π = 0.0055 and 0.0112, respectively) representing a 10% or a 20% decline over 20 years, respectively.

To explore the effect that the initial probability of occupancy (*ψ*_*1*_) would have on the power to detect trends in occupancy over time, we chose three values of *ψ*_*1*_. Because we were interested in the effect of lower values of *ψ*_*1*_ than were observed by Wallace et al. [27], we chose their reported mean of ${\hat{\psi }}_{1}$ = 0.72 (95% CI: 0.57–0.83) for 2011 as the highest value for our simulations. We chose the lower limit of the 95% CI of ${\hat{\psi }}_{1}$ in 2011 of 0.57 as a middle value for *ψ*_*1*_. The lowest value for *ψ*_*1*_ we selected was 0.42, which represented the same distance from the lower limit of the 95% CI of *ψ* in 2011 (i.e., 0.57–0.15 = 0.42) as the lower limit was from the mean (i.e., 0.72–0.57 = 0.15).

We estimated the average probability of colonization for season *i* (*γ*_*i*_) as:

$$\gamma_{i}=\frac{(\pi +\varepsilon )∙\psi_{i}}{\left(1-\psi_{i}\right)}.$$

The probability that a site was occupied in the first season (*ψ*_*1*_) was a parameter defined for a simulation. The probability that a site was occupied in subsequent seasons was given as:

$$\psi_{i}=\psi_{i-1}*(1-\varepsilon )+(1-\psi_{i-1})*\gamma_{i-1}$$

For season *i* we determined if a site was occupied using a random Bernoulli draw with probability *ψ*_*i*_. If the site was determined to be occupied based on the Bernoulli draw, we determined if occupancy was detected using random additional draws from a binomial distribution with probability *p* and the number of trials equal to the number of visits. In each simulation run, we fit a multi-season occupancy model to the simulated data to estimate the average annual percent decline in the population. During preliminary simulations we used program GENPRES [38] and PRESENCE [47] but determined that it did not provide the flexibility or full suite of metrics we used to evaluate trends (description below). We used the *unmarked* package [48] in the R language and environment (v3.2.1; R Development Core Team 2015) to fit a multi-season occupancy model to the simulated data. We estimated a trend parameter for probability of colonization while initial probabilities of occupancy, detection, and extinction were estimated without covariates. We used Markov model smoothing to estimate annual occupancy, which obtained predictions from the multi-season occupancy model conditional on the observed data [48]. Finally, we regressed annual smoothed occupancy estimates from the multi-season occupancy model on year using log-linear regression, and then used the slope parameter from the regression model as an estimate of trend (i.e., annual change in occupancy rate).

Reliable trend estimation may require monitoring to extend for a relatively long time period. Long-lived species that have high site fidelity, like ferruginous hawks, might require longer time periods for changes in occupancy to manifest in obvious trends. Furthermore, a long-term data set might be required to separate true population trends from inter-annual variation inherent to populations [49]. To evaluate the ability of a monitoring program to detect population declines with respect to the total duration of monitoring, we simulated monitoring for 10 and 20 years. Although understanding trends in occupancy in fewer years would be desirable, the number of years provides the number of data points from which a trend may be estimated; thus, we evaluated the minimum number of years we expected may provide enough information for a robust trend estimation.

#### Metrics to evaluate simulations

We used four metrics to evaluate how sampling effort and occupancy parameters affected the ability to detect trends in site occupancy: relative bias (RBIAS), coefficient of variation (CV), confidence interval (CI) coverage, and statistical power. Using multiple metrics is preferable because evaluating a single metric can be misleading; when selecting an appropriate monitoring design, understanding the precision and bias associated with the monitoring effort are as important as understanding statistical power.

We measured RBIAS as the difference between the estimated trend and the true trend standardized by the true trend:

$$RBIAS=\frac{\sum (\hat{T}-T)/T}{I}∙100$$

where $\hat{T}$ was the estimated trend and *T* was the true simulated trend, and *I* = 1,000 (number of iterations in the simulation). Relative bias is interpreted as the magnitude of the difference between the estimated and actual trend, in terms of the true trend [50]. We measured CV as the median of the ratio of the standard deviation of the estimated trend to the mean of the estimated trend over 1,000 iterations in each simulation. We evaluated CI coverage as the percentage of 1,000 simulations resulting in 90% CIs that contained the true trend (*T*). Finally, we evaluated statistical power, or the proportion of times the analyses correctly detected a trend when one existed (i.e., correct rejection of a null hypothesis of no trend).

Strict guidelines on acceptable benchmarks for these metrics are not available, but a goal for this analysis was to evaluate the performance of particular monitoring scenarios to provide guidance on a monitoring program for ferruginous hawks. Thus, we selected conservative benchmark values for each metric to help identify monitoring effort appropriate for this species (Table 2). Any non-zero value of relative bias indicates a biased estimate, so we chose a low absolute value of ≤5% as acceptable. A lower CV percentage indicates higher precision of the estimated population trend; thus, we considered CVs ≤ 20% as an acceptable level of uncertainty. Ideally, a 90% CI should contain the true trend at least 90% of the time, so we selected a benchmark of 90% as a reference value for CI coverage. For statistical power, we used the conventional value of 80% as an acceptable benchmark [51–53]. However, our choice of benchmarks for all metrics are not meant to be prescriptive, and the desired levels of relative bias, CV, CI coverage, and power for a particular monitoring program should be decided upon after evaluating the context within which the program takes place, and weighing the relative costs and benefits of either detecting a trend when one does not exist or not detecting a trend when one exists [51].

**Table 2:** Metrics and associated benchmark values used to evaluate simulations of territory-occupancy monitoring for ferruginous hawks.

Metric to evaluate simulation	Benchmark value^a^
Relative bias	≤ 5%
Coefficient of variation	≤ 20%
Confidence interval coverage	90%
Statistical power	80%

^a^ See text for justification of benchmark values.

## Results

The plausibility of a removal design was of primary interest given the logistical and financial difficulties of monitoring raptors at broad spatial scales. Thus, we present results from the removal design first, followed by results from a standard sampling design for comparison. Furthermore, we concentrate on results from 20 years of monitoring, assuming the long-term data would provide the most robust estimates of occupancy trend, followed by results from 10 years of monitoring to address whether trends could be detected in a shorter time frame.

### Survey effort

**Fig 1:**
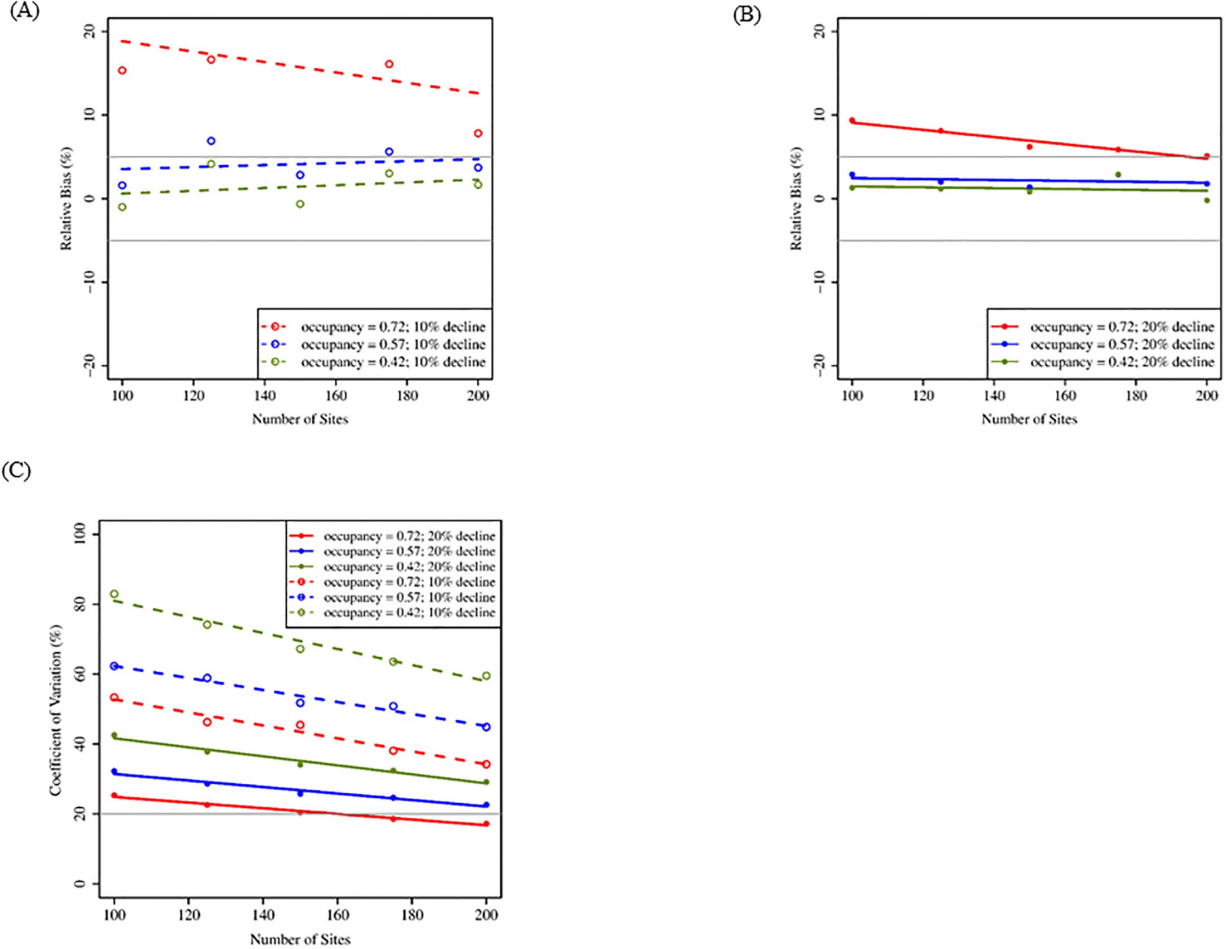
Mean relative bias and coefficient of variation for simulations increasing number of sites (removal design; 20 years). Mean relative bias (A: 10% decline; B: 20% decline) and coefficient of variation (C) for 100–200 sites, three initial occupancy rates, and two rates of population decline following a 20-yr period, assuming a maximum of three visits and a probability of detection = 0.67. These results reflect removal sampling in which territories are not visited after they are confirmed to be occupied within a given breeding season. Gray lines indicate benchmark values for respective metrics (Table 2).

Using the removal sampling design, our simulations suggested that increasing the number of sites from 100 to 200 resulted in largest improvements of metrics related to precision and statistical power, but the magnitude of improvement was dependent on rates of occupancy and size of population decline. Relative bias remained < 20% as the number of sites increased regardless of initial occupancy rate and rate of population decline. When occupancy was high (0.72), improvements in RBIAS were larger as number of sites increased but was outside our acceptable benchmark of 5% even at the greatest number of sites (200; Fig 1). At lower occupancy rates, as the number of sites increased, RBIAS remained <10%, and in many cases ≤5% (Fig 1). Precision increased for all rates of occupancy and population decline as the number of sites increased from 100 to 200 (Fig 1). Confidence interval coverage ranged from 78–86% depending on rates of occupancy and population decline but was relatively constant with increasing number of sites (Table 3). Statistical power ranged from 44.3–99.9% and improved as the number of sites increased from 100 to 200 (Table 3).

**Table 3:** Evaluation metrics for simulations increasing number of sites monitored.

Monitoring design; total duration	Metric	Number of sites	Probability of occupancy = 0.42		Probability of occupancy = 0.57		Probability of occupancy = 0.72	
			10% decline	20% decline	10% decline	20% decline	10% decline	20% decline
Removal; 20 yrs	Confidence interval coverage (%)	100	87.9	88.1	88.3	87.7	79.7	77.7
		125	87.6	89.0	88.9	88.8	76.6	80.8
		150	89.4	88.7	87.4	89.1	70.5	83.9
		175	88.6	88.6	88.4	87.1	73.6	87.2
		200	87.4	89.2	87.9	87.5	87.3	86.7
	Statistical power (%)	100	44.3	**83.0**	59.9	**95.6**	69.2	**97.9**
		125	50.1	**89.7**	63.7	**98.0**	78.5	**99.5**
		150	57.4	**92.2**	72.0	**98.9**	78.0	**99.7**
		175	58.1	**95.6**	72.2	**99.6**	**84.4**	**99.4**
		200	61.8	**97.1**	79.2	**99.9**	**92.3**	**99.6**
Standard; 20 yrs	Confidence interval coverage (%)	100	**90.1**	86.3	88.6	88.8	86.9	88.1
		125	89.4	89.1	88.2	87.2	89.0	87.3
		150	89.3	89.8	87.1	87.0	87.1	85.9
		175	**90.0**	89.2	88.2	88.7	88.2	87.7
		200	**90.9**	88.6	87.3	88.5	86.2	86.5
	Statistical power (%)	100	43.8	**82.0**	58.6	**94.3**	73.0	**99.3**
		125	51.1	**89.0**	65.8	**97.6**	**84.1**	**99.9**
		150	53.9	**91.0**	71.0	**99.1**	**87.3**	**99.9**
		175	57.9	**96.6**	74.9	**99.8**	**90.8**	**100.0**
		200	63.3	**97.6**	**80.7**	**99.8**	**91.3**	**100.0**
Standard; 10 yrs	Confidence interval coverage (%)	100	88.3	87.3	89.1	85.5	84.6	86.7
		125	86.3	88.4	87.3	86.5	86.7	86.3
		150	87.7	86.9	87.3	88.7	85.3	85.8
		175	86.1	87.0	85.9	87.3	87.3	86.3
		200	88.1	87.2	88.4	87.7	87.3	88.1
	Statistical power (%)	100	19.1	34.4	23.4	43.7	34.4	61.1
		125	24.0	41.3	28.3	51.2	35.5	63.9
		150	27.3	40.7	25.9	55.8	38.9	71.5
		175	26.3	43.0	29.8	58.5	37.5	78.3
		200	25.0	46.2	33.6	60.7	41.2	**80.6**

Confidence interval coverage (%) and statistical power (%) for 100–200 sites, three initial occupancy rates, and two rates of population decline following 20-yr and 10-yr monitoring periods, and assuming a maximum of three visits/breeding season and probability of detection = 0.67. Results from the removal design reflect monitoring for which territories are not visited after they are confirmed to be occupied within a given breeding season; results from the standard design reflect monitoring for which all territories are visited an equal number of times within a season. Bold font indicates values that met or exceeded benchmarks used to evaluate simulations (i.e., ≥90% confidence interval coverage and ≥80% statistical power; Table 2).

**Fig 2:**
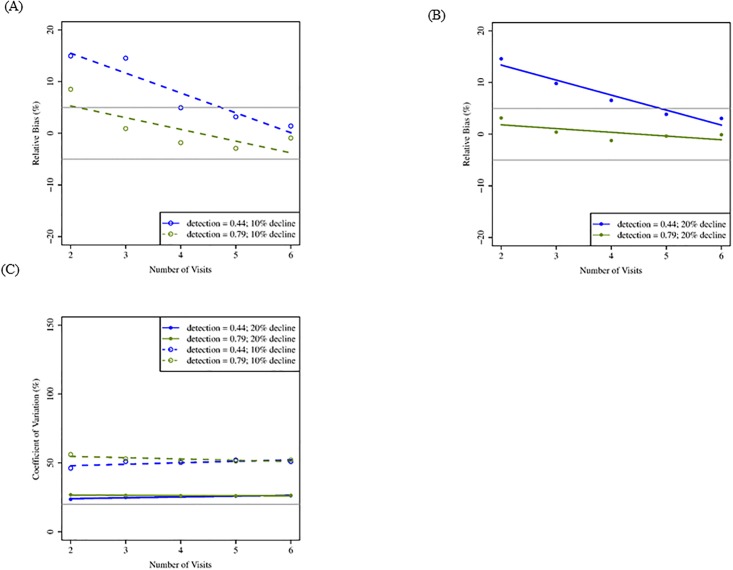
Mean relative bias and coefficient of variation for simulations increasing number of visits (removal design; 20 years). Mean relative bias (A: 10% decline; B: 20% decline) and coefficient of variation (C) for a maximum of two to six visits, two detection rates, and two rates of population decline following a 20-yr monitoring period assuming 150 sites, and initial probability of occupancy = 0.57. These results reflect removal sampling in which territories are not visited after they are confirmed to be occupied within a given breeding season. Gray lines indicate benchmark values for respective metrics (Table 2).

Using the removal sampling design, our simulations suggested that increasing the number of visits resulted primarily in improvements of bias and precision. As the maximum number of visits increased from two to six, RBIAS decreased (Fig 2). Relative bias was acceptable at two or three visits, generally under conditions of high detection rate (0.79). We did not observe changes in CV as the maximum number of visits increased from two to six, and CV was >20% when number of sites was held to 150, suggesting greater gains in precision of trend estimation would be achieved through the addition of more sites and not more visits (Fig 2). Similarly, CI coverage was sensitive to increasing number of visits but remained below our acceptable threshold (90%) when detection was low (0.44; Table 4), suggesting more sites would need to be monitored to improve precision in trend estimation under these conditions. When detection was high (0.79), confidence interval coverage was generally near our acceptable level of 90%, but only when population declines were larger (20%; Table 4). Power was acceptable at ≥80% at two visits regardless of detection rate, but only when population decline was larger (20%; Table 4).

**Table 4:** Evaluation metrics for simulations increasing number of visits to sites.

Monitoring design; total duration	Metric	Number of visits per season	Probability of detection = 0.44		Probability of detection = 0.79	
			10% decline	20% decline	10% Decline	20% Decline
Removal; 20 yrs	Confidence interval coverage (%)	2	69.5	63.7	82.1	89.0
		3	79.6	79.1	88.4	91.5
		4	85.0	83.8	**90.1**	**90.4**
		5	88.6	88.3	89.5	89.3
		6	88.8	88.6	89.3	**91.6**
	Statistical power (%)	2	70.4	**96.3**	65.5	**98.8**
		3	68.2	**97.6**	71.2	**99.6**
		4	70.4	**98.9**	73.7	**99.5**
		5	73.2	**98.7**	72.6	**99.0**
		6	70.6	**99.0**	71.3	**98.9**
Standard; 20 yrs	Confidence interval coverage (%)	2	66.7	66.3	87.7	87.3
		3	80.5	78.3	89.8	89.0
		4	82.8	85.1	89.9	86.9
		5	88.7	85.9	**90.3**	**90.0**
		6	86.9	88.6	88.9	89.2
	Statistical power (%)	2	65.1	**96.4**	72.8	**98.7**
		3	72.8	**98.6**	73.7	**99.2**
		4	70.8	**97.7**	72.2	**99.4**
		5	68.8	**98.3**	69.5	**99.0**
		6	73.0	**98.6**	74.5	**99.5**
Standard; 10 yrs	Confidence interval coverage (%)	2	68.5	67.7	87.6	86.8
		3	80.3	81.4	87.5	87.8
		4	85.5	84.2	89.6	86.2
		5	86.6	87.5	89.9	88.7
		6	87.8	87.2	87.0	88.5
	Statistical power (%)	2	39.2	54.2	30.7	54.2
		3	33.4	53.9	26.5	54.2
		4	32.6	53.0	28.1	55.3
		5	27.6	52.7	28.7	53.2
		6	31.0	53.0	28.4	54.7

Confidence interval coverage (%) and statistical power (%) for a two to six visits per site, two detection rates, and two rates of population decline following 20-yr and 10-yr monitoring periods, assuming 150 sites and initial probability of occupancy = 0.57. Results from the removal design reflect monitoring for which territories are not visited after they are confirmed to be occupied within a given breeding season; results from the standard design reflect monitoring for which all territories are visited an equal number of times within a season. Bold font indicates values that met or exceeded benchmarks used to evaluate simulations (i.e., ≥90% confidence interval coverage and ≥80% statistical power; Table 2).

**Fig 3:**
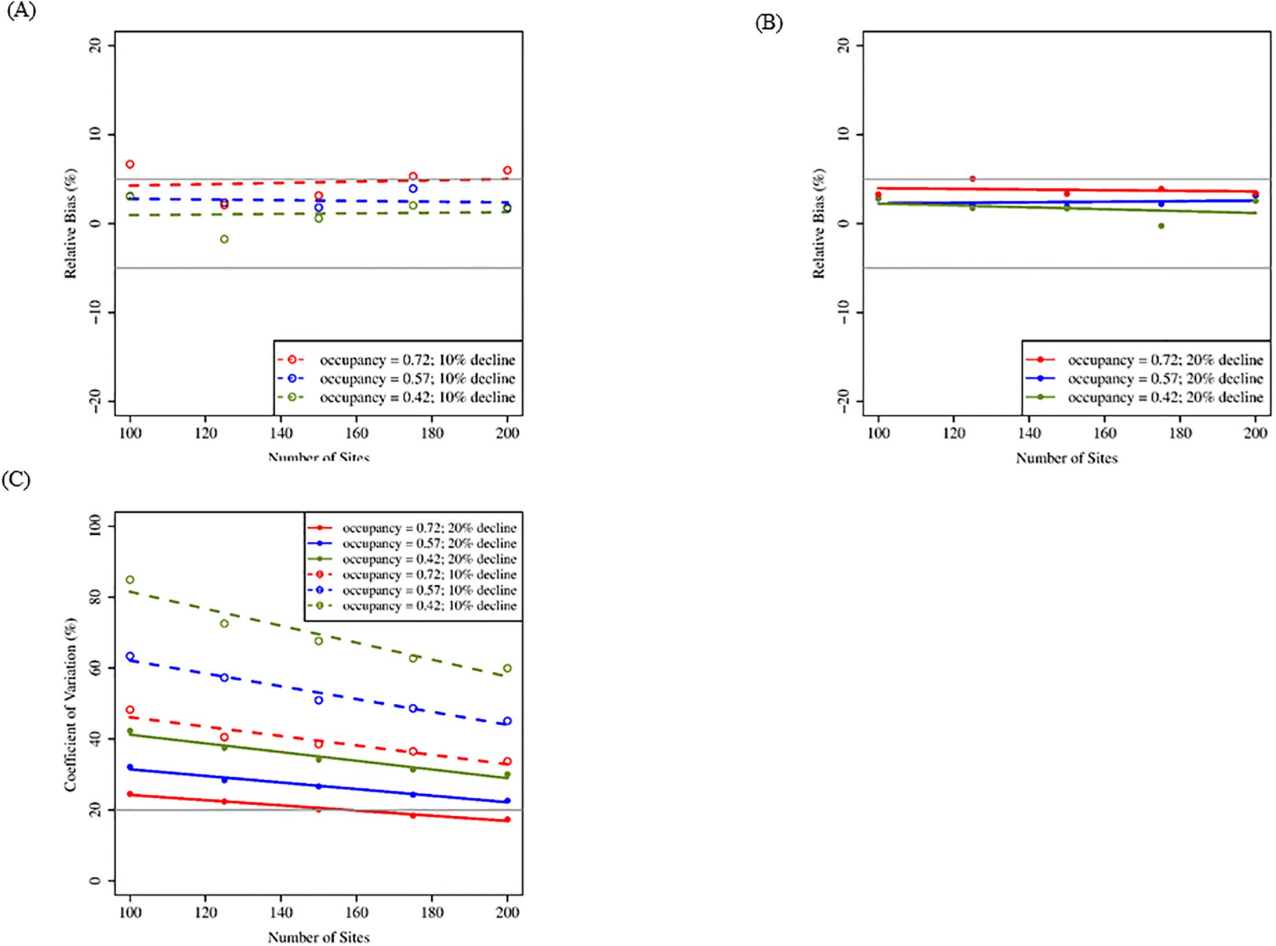
Mean relative bias and coefficient of variation for simulations increasing number of sites (standard design; 20 years). Mean relative bias (A: 10% decline; B: 20% decline) and coefficient of variation (C) for 100–200 sites, three initial occupancy rates, and two rates of population decline following a 20-yr monitoring period, assuming three visits and a probability of detection = 0.67. These results reflect a standard sampling design in which all territories are visited an equal number of times within a season. Gray lines indicate benchmark values for respective metrics (Table 2).

For the standard sampling design, increasing number of sites resulted in greatest improvement of precision and statistical power. Relative bias was < 10% and often < 5%; increasing the number of sites from 100 to 200 did not improve RBIAS under scenarios we evaluated (Fig 3). Increasing the number of sites decreased CV; however, CV did not approach the 20% benchmark until there were ~150 sites monitored, and only under conditions of high initial occupancy rate (0.72) and larger population decline (20%; Fig 3). Increasing the number of sites did not improve CI coverage, which was close to 90% under most conditions (Table 3). Increasing the number of sites increased statistical power to varying degrees depending on the scenario (Table 3). When population decline was larger (20%), 100 sites provided > 80% power, but when population decline was smaller (10%), 80% power was only achieved at relatively high initial occupancy (0.57 and 0.72; Table 3).

**Fig 4:**
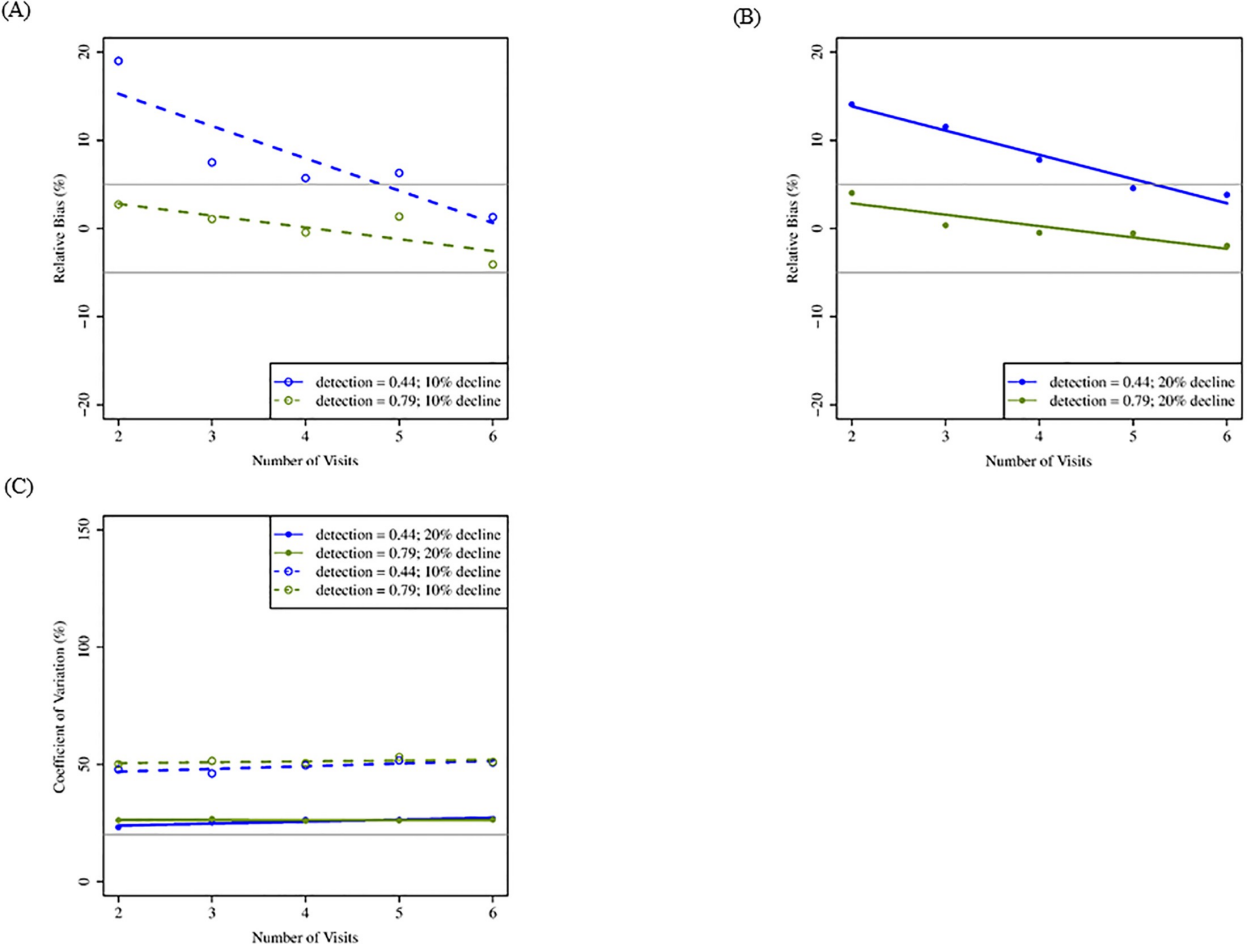
Mean relative bias and coefficient of variation for simulations increasing number of visits (standard design; 20 years). Mean relative bias (A: 10% decline; B: 20% decline) and coefficient of variation (C) for two to six visits to each site, two detection rates, and two rates of population decline following a 20-yr monitoring period, assuming 150 sites and initial probability of occupancy = 0.57. These results reflect a standard sampling design in which all territories are visited an equal number of times within a season. Gray lines indicate benchmark values for respective metrics (Table 2).

For the standard sampling design, increasing number of visits resulted in greatest improvement of bias and precision. Increasing from two to six visits reduced RBIAS most when the rate of detection was lower (0.44; Fig 4). Increasing number of visits did not influence CV (Fig 4) but did improve CI coverage when rate of detection was low (although our 90% benchmark was not achieved unless detection was higher; Table 4). Increasing number of visits improved statistical power most when detection was low and population decline small (10%), but power was >80% only when population decline was larger (20%; Table 4).

### Total duration of monitoring

**Fig 5:**
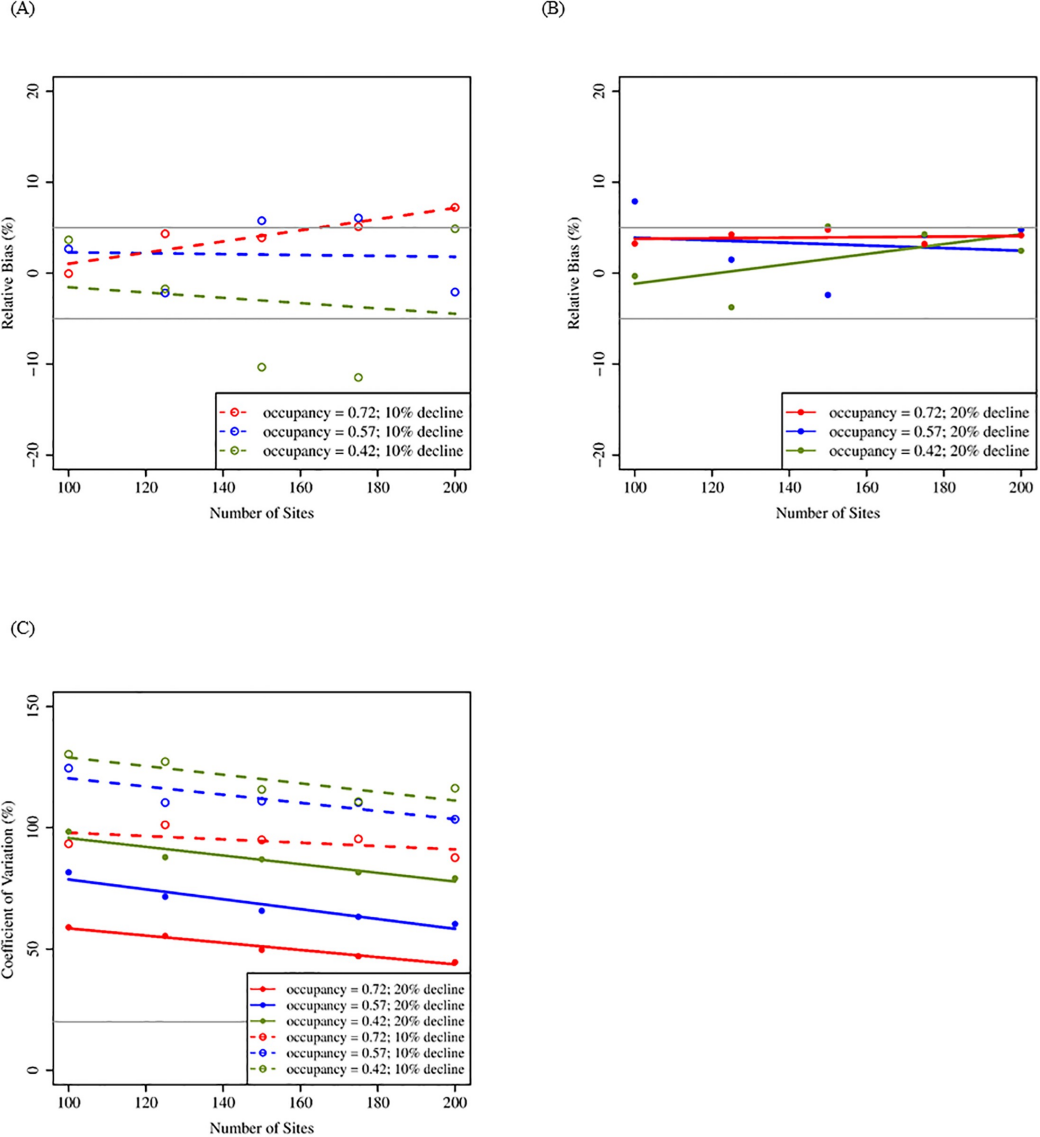
Mean relative bias and coefficient of variation for simulations increasing number of sites (standard design; 10 years). Mean relative bias (A: 10% decline; B: 20% decline) and coefficient of variation (C) for 100–200 sites, three occupancy rates, and two rates of population decline following a 10-yr monitoring period, assuming three visits and a probability of detection = 0.67. These results reflect a standard sampling design in which all territories are visited an equal number of times within a season. Gray lines indicate benchmark values for respective metrics (Table 2).

**Fig 6:**
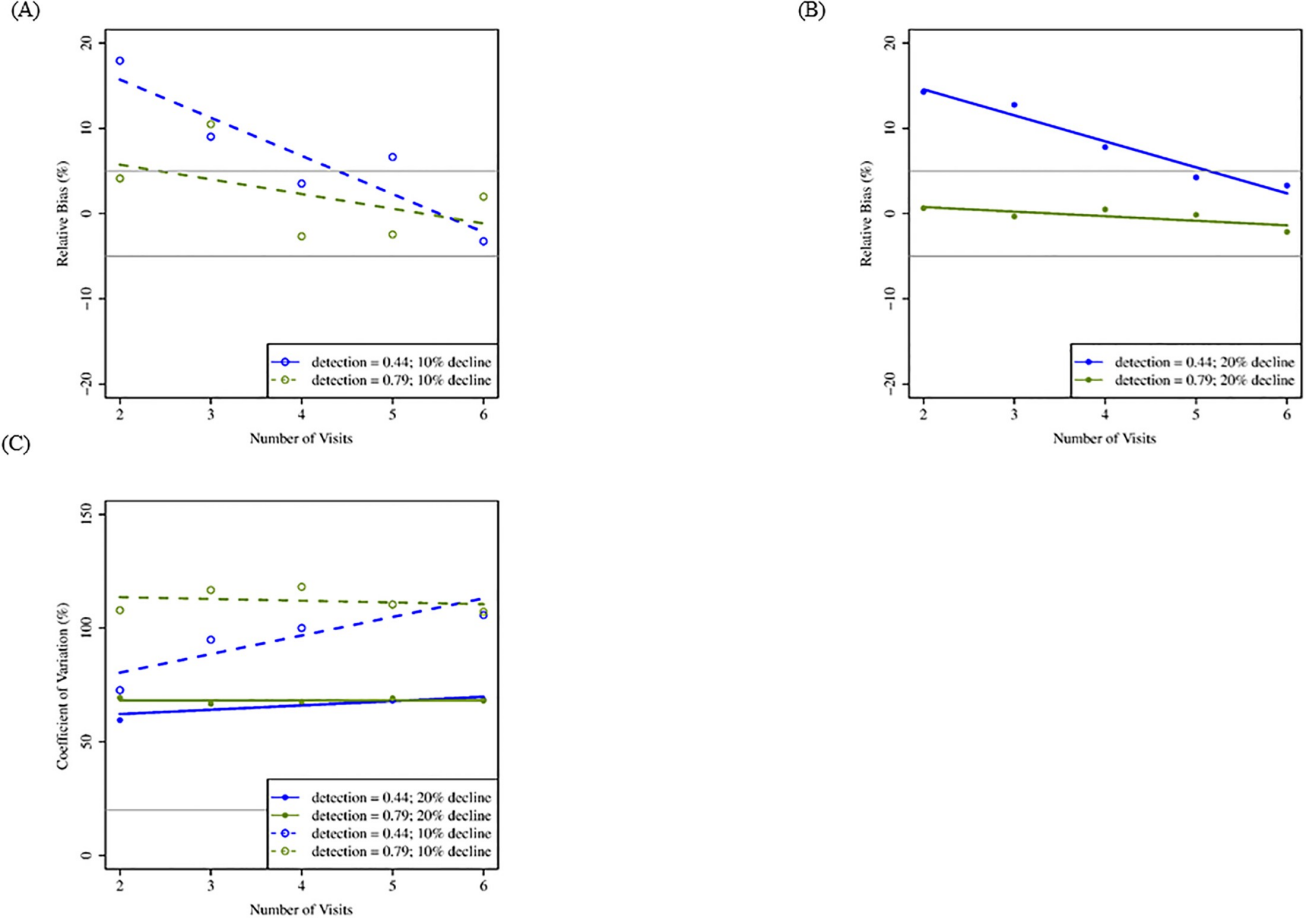
Mean relative bias and coefficient of variation for simulations increasing number of visits (standard design; 10 years). Mean relative bias (A: 10% decline; B: 20% decline) and coefficient of variation (C) for two to six visits to each site, two detection rates, and two rates of population decline following a 10-yr monitoring period, assuming 150 sites and initial probability of occupancy = 0.57. These results reflect a standard sampling design in which all territories are visited an equal number of times within a season. Gray lines indicate benchmark values for respective metrics (Table 2).

For the standard sampling design, the number of years of monitoring (10 vs. 20) had the largest effect on precision and statistical power. There was no clear difference in patterns of RBIAS (Figs 3–6). Coefficient of variation was generally lower when monitoring extended 20 years (Figs 3–6), but did not approach our benchmark of 25% unless population declines were larger (20%). Confidence interval coverage was not affected by duration of monitoring (Tables 3 and 4). There were large reductions in the power to detect a trend when monitoring occurred for only 10 years; 80% power was only reached when rate of occupancy was high (0.72), population declines were larger (20%), and the number of sites was at the highest value (200; Table 3). The 80% threshold was not reached by increasing the number of visits from two to six and was not improved when the probability of detection was high (0.79; Table 4). However, statistical power was ≥ 80% when monitoring extended for 20 years and population declines were larger (20%; Tables 3 and 4).

### Observation process

#### Probability of detection

For the removal design, lower probability of detection (0.44) generally resulted in higher values of RBIAS (Fig 2). Probability of detection did not influence CV across the range of number of visits (Fig 2) but did affect CI coverage (Table 4). Probability of detection affected statistical power less than rates of population decline, and power was acceptable (≥80%) when rate of population decline was greater (20%) at both detection rates (Table 4).

For the standard sampling design, detection probability had similar effects as the removal design on all four evaluation metrics. Lower detection probability (0.44) resulted in higher RBIAS, but RBIAS approached our acceptable level of 5% at 4–5 visits (Figs 4 and 6). Higher detection probability (0.79) resulted in acceptable levels of RBIAS at two visits. Probability of detection did not influence CV when number of visits increased for 20 years of monitoring (Fig 4), but for 10 years, CV was affected by number of visits when detection rate was lower (Fig 6). Probability of detection affected CI coverage; when detection was high (0.79), fewer visits were required to meet the acceptable level of 90% (Table 4). Probability of detection affected statistical power less than rates of population decline (Table 4).

### Occupancy parameters

#### Initial occupancy

For the removal sampling scheme, initial occupancy primarily affected measures of precision and statistical power. Initial occupancy influenced RBIAS but only when occupancy was high (Fig 1). For all occupancy rates, RBIAS approached our benchmark value (≤5%) once 150 sites were sampled when population decline was larger (Fig 1). Generally, higher initial occupancy resulted in lower CV, and CV decreased with the addition of more sites regardless of occupancy probability. However, the acceptable threshold for CV (20%) was not met unless occupancy was high (0.72; Fig 1). Lower occupancy probabilities (0.42 and 0.57) had better CI coverage than high occupancy (0.72; Table 3). Initial occupancy affected the power to detect a trend at 100 sites and affected gains in power as the number of sites increased (Table 3).

For the standard sampling scheme, initial probability of occupancy primarily affected precision and statistical power. Initial probability of occupancy had almost no effect on RBIAS for 20 years of monitoring, which remained acceptable at all three occupancy probabilities (Fig 3) but did influence RBIAS under the 10-year scenario (Fig 5). Higher occupancy resulted in lower CV, and CV decreased with the addition of more sites regardless of occupancy. However, the acceptable level for CV (20%) was not met unless occupancy was high (0.72) and monitoring extended for 20 years (Fig 3). Initial probability of occupancy did not strongly affect CI coverage, which approached the 90% benchmark (>85%) for all occupancy probabilities (Table 3). Initial occupancy probability affected the power to detect a trend at 100 sites and affected gains in power as the number of sites increased (Table 3).

#### Population decline

Under the removal sampling design, rate of population decline had the greatest effect on precision and statistical power. Population decline had less influence on RBIAS than the probability of occupancy (Fig 1). However, higher rates of population decline resulted in lower RBIAS when occupancy was high (0.72; Fig 1). Coefficient of variation was strongly affected by rate of population decline (Figs 1 and 2). Greater (20%) population decline resulted in smaller CV than smaller (10%) population decline. Rate of population decline influenced CI coverage when occupancy rates were high (0.72; Table 3) and when maximum number of visits was low (2; Table 4). The statistical power to detect a trend in occupancy was up to 1.5 times better when population decline was greater (20%) but the 80% benchmark was rarely met under any sampling condition when population decline was lower (10%; Tables 3 and 4).

For the standard sampling design, rate of population decline had little effect on changes in RBIAS when increasing the number of sites (Figs 3 and 5). Patterns were similar for estimates of CV as for the removal design, and greater population declines (20%) resulted in greater precision (Figs 3–6). Rate of population decline influenced CI coverage even less than it did under the removal sampling design (Tables 3 and 4). The statistical power to detect a trend in occupancy was better when population decline was greater (20%) and was consistently at or above our benchmark value of 80% for 20 years of monitoring but not 10 years (Tables 3 and 4).

## Discussion

We demonstrated through simulation that a site-occupancy framework for monitoring ferruginous hawks or other territorial, low-density raptors has the power to detect population declines as small as 10% after a 20-year period under certain conditions of site occupancy and detection rates, many of which have empirical justification. Our results further the science of species monitoring by demonstrating the impact of decisions regarding trade-offs between number of sites and number of visits, and how these trade-offs are influenced by demographic parameters. Similar to Barata et al. [54], our simulations suggest that even when a survey design provides reasonable precision and statistical power, only moderate to large declines in occupancy will be detected, and smaller declines may require up to twice as many years of data to detect.

Any site-based monitoring program will be affected by three general types of influence: survey effort, such as the number of sites to be monitored, number of visits to a site, and total duration of the monitoring program; the observation process, which is affected by the probability of detection, and demographic parameters related to occupancy such as change in regional population size and associated population dynamics, and initial occupancy rates. We have shown that demographic rates influence the ability of a particular suite of sampling parameters to detect changes in occupancy. Thus, over the course of a 20-yr monitoring period, a monitoring program with fixed sampling parameters will vary in its ability to detect population trends.

### Survey effort

The trade-off between number of sites and number of visits should be evaluated when deciding on the structure of a monitoring program for raptors. In our study, increasing the number of sites improved nearly every metric we used to evaluate trend estimation, whereas increasing the number of visits primarily improved CI coverage and RBIAS (but not after 3–4 visits within a season). This was not surprising: increasing replication of sites has previously been reported to improve the ability to detect population trends [55]. Including at least 150 sites would provide reasonable estimates of trend in occupancy given the levels of other variables we simulated, but only for population declines of ≥20%. Sampling designs often lack the power to detect small changes in estimated parameters, but even a 20% population decline was not detectable with 80% power unless 200 sites were monitored for ten years (assuming high initial occupancy rates), or 100 sites for 20 years. If there is a need to offset the cost of additional sites, a removal sampling design that allows for up to four visits to a site within a season could be used without a loss of statistical power while providing acceptable levels of precision and bias. In fact, removal sampling did not result in substantial changes of any metrics we used to evaluate simulations, providing little justification for employing the standard design if occupancy is the sole variable of interest. In previous evaluations removal designs have been suggested to be less robust than standard designs because of limitations on evaluating variation in detection probability [47]. Although continued visits after occupancy is confirmed provide additional information on detection probability, removal designs may be beneficial because they reduce effort and associated cost of surveys as the total number of visits are fewer relative to a standard sample design and reduced mean effort per site might allow for a greater number of total sites to be surveyed within a given season [47]. However, the logistics of a removal-based survey may be more complicated as the actual number of visits to each site is unknown *a priori*, although the maximum number of visits to a given site may be pre-determined based on cost or other constraints. In years with lower occupancy and detection rates, costs could be greater than expected because more visits would be required, potentially approaching the effort for a standard design. Also, the logistics of adapting schedules and routes during the season to re-survey only sites without detections requires more planning than a standard survey design. If occupancy or detection rates are expected to be low, or if reproductive success is an additional variable of interest (thus requiring visits to a territory later in the breeding season), then a standard survey design is likely the most appropriate.

The total duration of monitoring influenced the power to detect trends in occupancy more strongly than any other variable related to survey effort, with a 20-yr monitoring program resulting in much more reliable trend estimates. The effect of temporal scale that we observed on the ability to detect a trend is not unusual; 10–20 years has been identified in other studies as the minimum duration of monitoring required to detect population trends for many species with any reasonable statistical power and depending on the amount of variation in the sample [56–58]. However, the total duration of monitoring required for reliable trend estimation is sensitive to sudden shifts in the data, which can occur when there are abrupt changes to the local environment (e.g., exceptional weather events, or habitat conversion near a territory). If raptor populations experience these “level shifts”, it may increase the number of years required to detect a trend by 50% or more [57].

### Observation process

Probability of detection is an important consideration when designing monitoring programs because of its influence on accuracy of estimates of population parameters [47]. Our simulations suggested higher detection probability resulted in improvements of trend detection; therefore, any monitoring program for raptors should benefit from designs that maximize the probability of detection. The probability of detection for nest or territory occupancy by raptors is usually < 1, and is influenced by factors including species, nesting phenology, nest characteristics, weather, observer experience, survey speed and duration, and aircraft type [30, 59, 27, 31]. Detection probability is also expected to vary depending on how sites are defined and selected; for example, detection rates may be lower for larger sites because they are more difficult to search thoroughly. As was the case with Wallace et al., [27] surveys should target time periods when detection probabilities are likely to be at their highest, which can vary among species because of differences in life history (e.g., long-distance migrants may initiate breeding activities later in the season than residents or short-distance migrants; e.g., [60]). In the study upon which our simulations were based, nest height was positively related to detection probability, and the relationship was especially strong in the first year of the study when observers had less knowledge of nest site locations [27]. It is likely that detection probabilities to determine raptor occupancy could be increased in similar studies by using experienced observers with knowledge of species-specific nest characteristics and breeding behavior.

### Occupancy parameters

Higher initial occupancy improved precision and power, a pattern observed in previous power analyses [61, 54]. It is possible that estimates of initial occupancy in our simulations are biased high because of the method used to build the sample of nests monitored by [27]. The locations of all stick nests were identified and mapped during an initial 2010–2011 stick nest survey, and a sample of these locations were subsequently evaluated for occupancy. This approach to monitoring raptor nests is not unusual: the low density of many raptor populations results in nest monitoring that is often focused on known (historic) nests or territories (e.g., [62, 63, 25–27, 31]), which ultimately restricts inference to patterns of re-use and does not allow evaluation of changes in distribution (specifically, colonization of previously unoccupied areas; 27]. Furthermore, the sample that represents a collection of previously-used sites potentially inflates the proportion of sites occupied in the sample relative to the proportion of sites occupied in the population, which can result in an estimated declining trend in occupancy when no such trend exists [33]. Thus, the data we have evaluated in our simulations likely represents a biased estimate of initial occupancy, but we do not know to what extent the sample may be biased. For this reason, quantifying the bias in the estimate of initial occupancy is not possible because we do not have “baseline” (i.e., true) occupancy rates to which we could compare. Evaluating bias using a method such as censoring the initial year of sampling from the data and subsequently estimating trends in occupancy may not address the effect of biased initial occupancy on trend estimation; we could not know if removing the first year, or the first and second year, etc. were effective at removing the bias. Furthermore, in practice, censoring initial years of data would reduce the number of years of data available for trend estimation, and would therefore only be feasible for retrospective analyses of longer-term monitoring data. We have addressed biased estimates of initial occupancy in our analysis by evaluating three different levels of initial site occupancy and its effect on the ability to detect trends in occupancy. By considering different values of initial probability of occupancy we have demonstrated how metrics change in cases where initial probability of occupancy is lower, so we have accounted for the potential effects of lower initial occupancy on a monitoring program.

Estimating site occupancy status over multiple breeding seasons allows estimation of local extinction and colonization, vital demographic rates by which species respond to changes in environmental conditions. Extinction and colonization rates may reflect changes in population size or may reflect changes in the spatial distribution of raptors (i.e., local immigration or emigration). Altered geographic distribution may be particularly important to consider in light of effects that changing landscapes and climate may impose upon raptor populations. As habitat continues to be converted and prey populations respond to changing environmental conditions, it is possible that raptors could colonize previously unused portions of their geographic ranges and shift their local and regional distributions. Some nest-based studies have addressed this issue by adding new nest sites or territories to samples as they are discovered [64, 27]; however, adding occupied sites to the sample population over the course of a study may introduce an unknown bias in trends. The spatial distribution of breeding raptors presumably is affected by the factors that drive productivity, in particular the abundance of prey [19, 27]. Human disturbance may also be a factor that influences extinction or colonization of breeding sites [65]. Thus, activities such as encroaching urban development, natural resource extraction, or other situations that result in increased human or vehicle activity or infrastructure in the vicinity of territories could influence colonization or extinction rates. These factors could be important habitat covariates that affect breeding site occupancy dynamics and should be considered when designing and implementing a monitoring program.

Although we present data from a study of ferruginous hawks, our simulations can be applied to other raptor species that may have similar life history and population dynamics to provide guidance for future surveys of territory occupancy rates and trend estimation. However, simulations are rarely complete (i.e., every possible combination of scenarios cannot reasonably be simulated), and multiple assumptions regarding initial probability of occupancy, detection, extinction, and annual rates of population change must be made. Thus, these simulations are meant to be a guide for developing monitoring programs and may need to be refined following additional data collection.

## References

[1] Millenium Ecosystem Assessment (US). Ecosystems and Human Well-Being: Synthesis. World Resources Institute, Washington, DC: Island Press; 2005.

[2] Boivin NL, Zeder MA, Fuller DQ, Crowther A, Larson G, Erlandson JM, et al Ecological consequences of human niche construction: examining long-term anthropogenic shaping of global species distributions. Proc Natl Acad Sci U S A. 2016; 113: 6388–6396. 10.1073/pnas.1525200113 27274046 PMC4988612

[3] Purvis A, Gittleman JL, Cowlishaw G, Mace GM. Predicting extinction risk in declining species. Proc R Soc Lond B 2000; 267: 1947–1952. 10.1098/rspb.2000.1234 PMC1690772 11075706

[4] International Union Conservation of Nature. The IUCN Red List of Threatened Species. Version 2017–2; 2017. http://www.iucnredlist.org. Accessed 14 September 2017.

[5] U.S. Fish and Wildlife Service (US). Birds of Conservation Concern 2008. Arlington, VA: United States Department of Interior, Fish and Wildlife Service, Division of Migratory Bird Management; 2008 85 p. https://www.fws.gov/migratorybirds/pdf/grants/BirdsofConservationConcern2008.pdf

[6] Helander B, Bignert A, Asplund L. Using raptors as environmental sentinels: monitoring the white-tailed sea eagle *Haliaeetus albicilla* in Sweden. Ambio 2008; 37: 425–431. 18833795 10.1579/0044-7447(2008)37[425:uraesm]2.0.co;2

[7] Lodenius M, Solonen T. The use of feathers of birds of prey as indicators of metal pollution. Ecotoxicology 2013; 22: 1319–1334. 10.1007/s10646-013-1128-z 24096904

[8] Sergio F, Caro T, Brown D, Clucas B, Hunter J, Ketchum J, et al Top predators as conservation tools: ecological rationale, assumptions, and efficacy. Annu Rev Ecol Evol Syst. 2008; 39: 1–19.

[9] Sergio F, Marchesi L, Pedrini P, Penteriani V. Coexistence of a generalist owl with its intraguild predator: distance-sensitive or habitat—mediated avoidance? Anim Behav. 2007; 74: 1607–1616.

[10] Greeney HF, Meneses MR, Hamilton CE, Lichter-Marck E, Mannan RW, Snyder N, et al Trait-mediated trophic cascade creates enemy-free space for nesting hummingbirds. Sci Adv. 2015; 1: e1500310 10.1126/sciadv.1500310 26601258 PMC4643763

[11] Molina-López RA, Casal J, Darwich L. Causes of morbidity in wild raptor populations admitted at a wildlife rehabilitation centre in Spain from 1995–2007: a long-term retrospective study. PLoS One 2011; 6: e24603 10.1371/journal.pone.0024603 21966362 PMC3179465

[12] Lehman RN, Kennedy PL, Savidge JA. The state of the art in raptor electrocution research: a global review. Biol. Conserv. 2007; 136: 159–174.

[13] De Lucas M, Janss GFE, Whitfield DP, Ferrer M. Collision fatality of raptors in wind farms does not depend on raptor abundance. J Appl Ecol. 2008; 45: 1695–1703.

[14] Hager SB. Human-related threats to urban raptors. J Raptor Res. 2009; 43: 210–226.

[15] Farmer CJ, Goodrich LJ, Inzunza ER, Smith JP. Conservation Status of North America’s Birds of Prey. Pp. 303–420 in K.L. Bildstein, J.P. Smith, E. Ruelas I., and R.R. Veit (eds). State of North America’s Birds of Prey. Nuttall Ornithological Club and American Ornithologists. Union Series in Ornithology No. 3. Cambridge, Massachusetts, and Washington, D.C. 2008.

[16] Knick ST, Dobkin DS, Rotenberry JT, Schroeder MA, Vander Haegen WM, van Riper C III. Teetering on the edge or too late? Conservation and research issues for avifauna of sagebrush habitats. Condor 2003; 611–634.

[17] Askins RA, Chavez-Ramiez F, Dale BC, Haas CA, Herkert JR, Knopf FL, Vickery PD. Conservation of grassland birds in North America: understanding ecological processes in different regions. Ornithol. Monogr. 2007; 64: 1–46.

[18] White EM, Morzillo AT, Alig RJ. Past and projected rural land conversion in the US at state, regional, and national levels. Landsc Urban Plan. 2008; 89: 37–48.

[19] Bechard MJ, Schmutz JK. Ferruginous hawk (Buteo regalis) In: Poole A, editor. The birds of North America. Ithaca: Cornell Lab of Ornithology; 2017 10.2173/bna.ferhaw.02

[20] Woodbridge B, Hargis CD. Northern goshawk inventory and monitoring technical guide. Washington, DC: U.S. Department of Agriculture, Forest Service; 2006. 80 p. Gen. Tech. Rep. WO-71.: https://www.fs.fed.us/biology/wildecology/docs/GoshawkTechGuideJuly06.pdf

[21] U.S. Fish and Wildlife Service (US). Post-delisting Monitoring Plan for the Bald Eagle (Haliaeetus leucocephalus) in the Contiguous 48 States. Twin Cities, MN: U.S. Fish and Wildlife Service, Divisions of Endangered Species and Migratory Birds and State Programs, Midwest Regional Office; 2009. 75 p. https://www.fws.gov/midwest/eagle/protect/pdf/BEPDMP_100511_OMBFINALfor%20posting_Jan2013Final.pdf

[22] Pagel JE, Whittington DM, Allen GT. Interim Golden Eagle inventory and monitoring protocols; and other recommendations. Arlington, VA: Division of Migratory Bird Management, U.S. Fish and Wildlife Service; 2010 27 pp. https://www.fws.gov/southwest/es/oklahoma/documents/te_species/wind%20power/usfws_interim_goea_monitoring_protocol_10march2010.pdf

[23] Bart J, Buchanan JB, Altman B. Improving the Breeding Bird Survey. In: Ralph CJ, Rich TD, editors. Bird Conservation Implementation and Integration in the Americas: Proceedings of the Third International Partners in Flight Conference. California: USDA Forest Service; 2005. p. 771–776. Gen Tech Rep PSW-GTR-191.

[24] Anderson DE. Survey techniques In: Bird DM, Bildstein KL, editors. Raptor research and management techniques. Blaine, WA: Hancock House Publishers 2007 pp 89–100.

[25] Jiménez-Franco MV, Martínez JE, Calvo JF. Territorial occupancy dynamics in a forest raptor community. Oecologia 2011; 166: 507–516. 10.1007/s00442-010-1857-0 21136084

[26] Brown JL, Steenhof K, Kochert MN, Bond L. Estimating raptor nest success: old and new approaches. J Widl Manage. 2013; 77: 1067–1074. 10.1002/jwmg.566 PMC4577060 26401058

[27] Wallace ZP, Kennedy PL, Squires JR, Oakleaf RJ, Olson LE, Dugger KM. Re-occupancy of breeding territories by Ferruginous hawks in Wyoming: relationships to environmental and anthropogenic factors. PLoS One 2016; 11: e0152977 10.1371/journal.pone.0152977 27049324 PMC4822948

[28] Wiggins DA, Grzybowski JA, Schnell GD. Ferruginous hawk demography in areas differing in energy extraction activity. J Wildl Manage. 2017; 81: 337–341.

[29] Bird DM, Bildstein KL, editors. Raptor research and management techniques. Surrey, British Columbia: Hancock House Publishers; 2007.

[30] Booms TL, Schempf BF, McCaffery BJ, Lindberg MS, Fuller MR. Detection probability of cliff-nesting raptors during helicopter and fixed-wing aircraft surveys in western Alaska. J Raptor Res. 2010; 44: 175–187.

[31] Stahlecker DW, Wallace ZP, Mikesic DG, Smith CS. Does Hopi religious harvest of eaglets affect golden eagle territory occupancy and reproduction on the Navajo Nation? J Raptor Res. 2017; 51: 305–318.

[32] Lõhmus A. Are certain habitats better every year? A review and a case study on birds of prey. Ecography 2003; 26: 545–552.

[33] MacKenzie DI, Nichols JD, Royle JA, Pollock KH, Bailey LL, Hines JE. Occupancy estimation and modeling: inferring patterns and dynamics of species occurrence. Amsterdam: Elsevier/Academic Press; 2006.

[34] MacKenzie DI, Nichols JD, Lachman GB, Droege S, Royle JA, Langtimm CA. Estimating site occupancy rates when detection probabilities are less than one. Ecology 2002; 83: 2248–2255.

[35] Zielenski WJ, Stauffer HB. Monitoring *Martes* populations in California: survey design and power analysis. Ecol Appl. 1996; 6: 1254–1267.

[36] Bruggeman JE, Anderson DE, Woodford JE. Northern Goshawk monitoring in the western Great Lakes Bioregion. J Raptor Res. 2011; 45: 290–303.

[37] Keane A, Hobinjatovo T, Razafimanahaka HJ, Jenkins RKB, Jones JPG. The potential of occupancy modelling as a tool for monitoring wild primate populations. Anim Conserv. 2012; 15: 457–465.

[38] Bailey LL, Hines JE, Nichols JD, MacKenzie DI. Sampling design trade-offs in occupancy studies with imperfect detection: examples and software. Ecol Appl. 2007; 17: 281–290. 17479851 10.1890/1051-0761(2007)017[0281:sdtios]2.0.co;2

[39] Shannon G, Lewis JS, Gerber BD. Recommended survey designs for occupancy modelling using motion-activated cameras: insights from empirical wildlife data. PeerJ 2014; 2:e532; 10.7717/peerj.532 25210658 PMC4157302

[40] Ellis MM, Ivan JS, Schwartz MK. Spatially-explicit power analyses for occupancy-based monitoring of wolverine in the U.S. Rocky Mountains. Conserv Biol. 2014; 28: 52–62. 10.1111/cobi.12139 24001256

[41] Ellis MM, Ivan JS, Tucker JM, Schwartz MK. rSPACE: spatially-based power analysis for conservation and ecology. Methods Ecol Evol. 2015; 6: 621–625.

[42] Steenweg R, Whittington J, Hebblewhite M, Forshner A, Johnston B, Peterson D, et al Camera-based occupancy monitoring at large scales: power to detect trends in grizzly bears across the Canadian Rockies. Biol Conserv. 2016; 201: 192–200.

[43] Latif QS, Ellis MM, Saab VA, Mellen-McLean K. Simulations inform design of regional occupancy-based monitoring for a sparsely distributed, territorial species. Ecol Evol. 2017; 10.1002/ece3.3725 29375788 PMC5773320

[44] Lanier WE, Blakesley JA. Site Occupancy by Mexican Spotted Owls (Strix occidentalis lucida) in the US Forest Service Southwestern Region, 2015. Bird Conservancy of the Rockies. Brighton, Colorado, USA; 2015.

[45] Olson LE, Oakleaf RJ, Squires JR, Wallace ZP, Kennedy PL. Nesting pair density and abundance of ferruginous hawks (*Buteo regalis*) and golden eagles (*Aquila chrysaetos*) from aerial surveys in Wyoming. J Raptor Res. 2015; 49: 400–412.

[46] FGDC Cadastral Subcommittee PLSS Work Group Standardized PLSS Data Set(CADNSDI) Users Reference Materials. 2011 http://www.geocommunicator.gov/GeoComm/lsis_home/home/PLSS_CadNSDI_Publication_Handbook.pdf. Accessed 1 January 2013.

[47] MacKenzie DI, Royle JA. Designing occupancy studies: general advice and allocating survey effort. J Appl Ecol. 2005; 42: 1105–1114.

[48] Fiske I, Chandler RB. Unmarked: An R package for fitting hierarchical models of wildlife occurrence and abundance. J. Stat. Softw. 2011; 43; 1–23.

[49] Dodd CK. Monitoring amphibians in Great Smoky Mountains National Park. U.S.G.S. Report No. 1258: 2003; 118 p.

[50] Walther BA, Moore JL. The concepts of bias, precision and accuracy, and their use in testing the performance of species richness estimators, with a literature review of estimator performance. Ecography 2005; 28: 815–829.

[51] Di Stefano J. How much power is enough? Against the development of an arbitrary convention for statistical power calculations. Funct Ecol. 2003; 17: 707–709.

[52] Good RE, Nielson RM, Sawyer H, McDonald LL. A population estimate for golden eagles in the western United States. J Wildl Manage. 2007; 71: 395–402.

[53] Christensen P, Ringvall AH. Using statistical power analysis as a tool when designing a monitoring program: experience from a large-scale Swedish landscape monitoring program. Environ Monit Assess. 2013; 185: 7279–7293. 10.1007/s10661-013-3100-z 23377754

[54] Barata IM, Griffiths RA, Ridout MS. The power of monitoring: optimizing survey designs to detect occupancy changes in a rare amphibian population. Sci Rep. 2017; 7: 16491 10.1038/s41598-017-16534-8 29184083 PMC5705711

[55] Sauer JR, Fallon JE, Johnson R. Use of North American Breeding Bird Survey data to estimate population change for Bird Conservation Regions. J Wildl Manage. 2003; 67: 372–389.

[56] Hatfield JS, Gould WR IV, Hoover BA, Fuller MR, Lindquist EL. Detecting trends in raptor counts: power and Type I error rates of various statistical tests. Wildl Soc Bull. 1996; 24: 505–515.

[57] Weatherhead EC, Reinsel GC, Tiao GC, Meng XL, Choi D, Cheang WK, Keller T, DeLuisi J, Wuebbles DJ, Kerr JB, Miller AJ, Oltmans SJ, Frederick JE. Factors affecting the detection of trends: statistical considerations and applications to environmental data. J Geophys Res. 1998; 103: 17149–17161.

[58] Chambers LE, Patterson T, Hobday AJ, Arnould JPY, Tuck GN, Wilcox C, Dann P. Determining trends and environmental drivers from long-term marine mammal and seabird data: examples from Southern Australia. Reg Env Change. 2015; 15: 197–209.

[59] Murn C, Holloway GJ. Using areas of known occupancy to identify sources of variation in detection probability of raptors: taking time lowers replication effort for surveys. R Soc Open Sci. 2016; 3: 160368 10.1098/rsos.160368 27853552 PMC5098977

[60] Kennedy PL, Bartuszevige AM, Houle M, Humphrey AB, Dugger KM, Williams J. Stable occupancy by breeding hawks (Buteo sp.) over 25 years on a privately managed bunchgrass prairie in northeastern Oregon, USA. Condor 2014; 116: 435–445.

[61] Guillera-Arroita G, Lahoz-Monfort JJ. Designing studies to detect differences in species occupancy: power analysis under imperfect detection. Methods Ecol Evol. 2012; 3: 860–869.

[62] Kochert MN, Steenhof K, Carpenter LB, Marzluff JM. Effects of fire on golden eagle territory occupancy and reproductive success. J Wildl Manage. 1999; 63: 773–780.

[63] Olson GS, Anthony RG, Forsman ED, Ackers SH, Loschl PJ, Reid JA, Dugger KM, Glenn EM, Ripple WJ. Modeling of site occupancy dynamics for northern spotted owls, with emphasis on the effects of barred owls. J Wildl Manage. 2005; 69: 918–932.

[64] Dugger KM, Anthony RG, Andrews LS. Transient dynamics of invasive competition: barred owls, spotted owls, habitat, and the demons of competition present. Ecological Applications 2011; 21: 2459–2468. 22073635 10.1890/10-2142.1

[65] Jasikoff TM. Habitat suitability index models: Ferruginous hawk. U.S. Fish and Wildlife Service. FWS/OBS-82/10.10. 1982. 18 pp.

